# Toxicity Profile of *Plectranthus glandulosus* Hook. F. (Lamiaceae) Aqueous Extract, Hydro-Ethanolic Extract, and Ethyl Acetate Fraction

**DOI:** 10.1155/bri/7453400

**Published:** 2025-11-17

**Authors:** Djamila Zouheira, Moïse Legentil Nchouwet, Njini Gael Nfor, Hadidjatou Daïrou, Jean Romuald, Tigamba Vandi, Sylviane Laure Poualeu Kamani, Armelle Deutou Tchamgoue, Lauve Rachel Tchokouaha Yamthe, Sylvie Lea Wansi Ngnokam, Gabriel Agbor Agbor

**Affiliations:** ^1^Centre for Research on Medicinal Plants and Traditional Medicine, Institute of Medical Research and Medicinal Plants Studies, P.O. Box 13033, Yaoundé, Cameroon; ^2^Department of Animal Biology, Faculty of Sciences, University of Dschang, P.O. Box 67, Dschang, Cameroon; ^3^Department of Biological Sciences, Faculty of Science, University of Ngaoundere, P.O. Box 454, Ngaoundere, Cameroon

**Keywords:** acute toxicity, biochemical parameters, cytotoxicity, hematological parameters, *Plectranthus glandulosus*, subacute toxicity

## Abstract

*Plectranthus glandulosus* is valued for its culinary and medicinal properties. This study evaluated the cytotoxicity, acute oral toxicity, and subacute oral toxicity of its aqueous extract, hydro-ethanolic extract, and ethyl acetate fraction. Cytotoxicity was tested on African green monkey kidney (Vero) and murine macrophage (RAW 264.7) cell lines exposed to concentrations ≤ 200 μg/mL. Acute toxicity was assessed via a single oral dose of 5000 mg/kg, and subacute toxicity through daily oral doses of 250–1000 mg/kg over 28 days. All extracts maintained > 95% cell viability in Vero and RAW 264.7 cells, indicating no cytotoxicity. No mortality, behavioral changes, or clinical signs of toxicity were observed. Food and water intake, body weight, and organ weights were unaffected (*p* > 0.05). Macroscopic and histopathological examinations of the liver, kidneys, heart, and spleen showed no abnormalities. At the highest subacute dose (1000 mg/kg), hematological parameters remained within normal ranges, showing no significant differences compared to the control (*p* > 0.05): white blood cells (7.23–7.95 vs. 7.94 ± 1.29 × 10^3^/mm^3^), red blood cells (3.00–3.38 vs. 3.57 ± 0.79 × 10^6^/mm^3^), hemoglobin (12.10–12.40 vs. 12.20 ± 1.10 g/dL), hematocrit (29.60–44.53 vs. 41.70 ± 3.70%), and platelets (491.30–528.13 vs. 490.70 ± 0.40 × 10^3^/mm^3^). Differential leukocyte counts (lymphocytes, monocytes, eosinophils, and basophils) also remained stable. Biochemical markers, including alanine transaminase (62.76–69.20 vs. 64.60 ± 10.25 U/L), aspartate transaminase (112.15–123.85 vs. 120.69 ± 9.56 U/L), total protein (64.45–65.80 vs. 65.2 ± 3.20 g/L), urea (20.35–21.22 vs. 21.62 ± 0.73 mg/dL), creatinine (0.56–0.58 vs. 0.56 ± 0.33 mg/dL), and C-reactive protein (≤ 350 µg/mL), were comparable to controls. In conclusion, *P. glandulosus* extracts and fraction exhibited no cytotoxic, acute, or subacute toxic effects at the tested doses, supporting their safety and potential in alternative medicine.

## 1. Introduction

As part of their culture and customs, local and Indigenous groups have used plants for generations to prepare remedies to treat illnesses in humans and animals. Traditionally, people worldwide have used approximately 10,000 therapeutic plants of an estimated 250,000 higher plant species [1]. The World Health Organization (WHO) estimates that 65%–80% of people in developing countries rely on medicinal plants for primary health care [2], mainly due to the high cost and limited availability of synthetic medications [3]. In many regions, particularly in Africa, Asia, and South America, medicinal plants are readily accessible and often constitute the only source of health care for impoverished populations [4, 5]. Their use has developed through experience, and belief in the efficacy and safety of natural remedies, and a sense of empowerment in managing illnesses [6]. However, unregulated use and excessive dosages can cause harm; long-term consumption has been associated with organ toxicity [7]. Standardizing safety protocols is therefore essential to ensure high-quality, side-effect-free traditional medicines. Moreover, with increasing calls to integrate traditional medicine into modern healthcare systems [8], evaluating the potential toxicological risks of medicinal plants has become necessary [9].

*Plectranthus glandulosus*, a climbing herbaceous plant of the family Lamiaceae (Hook. f.), is distributed across West, Central, and Southern Africa. It is traditionally used to treat dermatitis, stomachaches, venereal infections, inflammation, nerve pain, cough, influenza, chest pain, and snake bites [10–13]. It also protects stored grains from pests, such as *Sitophilus zeamais* and *Callosobruchus maculatus* [14–17], and its leaves are used as a cooking condiment among the Ewondo people of Cameroon [18]. The plant contains secondary metabolites, including polyphenols, alkaloids, flavonoids, saponins, terpenoids, and tannins [19–25]. These compounds are likely responsible for its pharmacological activities, such as anti-inflammatory, antinociceptive, and antioxidant effects [22, 26, 27], as well as inhibition of low-density lipoprotein oxidation, lipid uptake, pancreatic lipase, cholesterol esterase, and thrombotic and atherogenic processes [23, 25, 28]. These activities are particularly relevant in combating cardiovascular diseases, a leading cause of mortality worldwide [29].

This study aimed to evaluate the potential toxicity of *P. glandulosus* leaves by investigating the cytotoxic effects of the aqueous and hydro-ethanolic extracts, as well as the ethyl acetate fraction, on cell lines. In addition, acute and subacute oral toxicities were assessed in Wistar rats to validate the plant's safety for regular culinary and therapeutic use.

## 2. Materials and Methods

### 2.1. Chemicals and Cell Lines

African green monkey normal kidney (Vero) cells (ATCC CRL-1586) and murine macrophage (RAW 264.7) cells (ATCC TIB-71) were obtained from the American Type Culture Collection (ATCC). The following reagents were purchased from Sigma-Aldrich Co. (St. Louis, MO, USA): Dulbecco's modified Eagle's Medium (DMEM), fetal bovine serum (FBS), sodium bicarbonate (NaHCO_3_), penicillin, streptomycin, trypsin, ethylenediaminetetraacetic acid (EDTA), podophyllotoxin, resazurin, phosphate-buffered saline (PBS), sodium chloride (NaCl), alanine aminotransferase (ALT), aspartate aminotransferase (AST), creatinine (Crea), urea, total protein (TP), C-reactive protein (CRP), hematoxylin, and eosin.

### 2.2. Plant Material

The mature, fully expanded green leaves of *Plectranthus glandulosus* were harvested from healthy plants in the Adamawa Region of Cameroon. The plant material was identified at the National Herbarium of Yaoundé, Cameroon, and a voucher specimen was deposited under the number 41168-HCN [22].

### 2.3. Preparation of *Plectranthus glandulosus* Leaves' Aqueous and Hydro-Ethanolic Extracts

The aqueous and hydro-ethanolic extracts of *Plectranthus glandulosus* were prepared following previously described methods [22, 28]. According to traditional healer recommendations, the aqueous extract of *P. glandulosus* leaves is traditionally prepared by macerating 20 g of powdered leaves in 166.6 mL of water, with a single dose administered daily for the treatment of obesity and high blood pressure. In this study, the aqueous extract was prepared in a larger batch by macerating 1200 g of powdered leaves in 10 L of distilled water.

After filtration, the filtrate was evenly distributed across several trays and placed on the shelves of a ventilated drying oven (MANESTY-PETERI SLW750) at 50°C for 24 h. The resulting yield was 11.15%. For the hydro-ethanolic extract, 2400 g of powdered leaves was macerated in a 70:30 (v/v) mixture of ethanol and distilled water (16.8 L ethanol and 7.2 L distilled water). Two successive macerations were performed, each using fresh solvent. The solvent was removed using a rotary evaporator (BUCHI), and the resulting extract was dried in a ventilated oven (MANESTY-PETERI SLW750) at 50°C for 24 h. The extraction yield was 10.96%. Both extracts were stored at −4°C until further use.

### 2.4. Preparation of *Plectranthus glandulosus* Leaves' Ethyl Acetate Fraction

As previously described, the hydro-ethanolic extract was further fractionated to obtain the ethyl acetate fraction [22]. Briefly, 190 g of the hydro-ethanolic extract was dissolved in 500 mL of distilled water and transferred to a separating funnel. Subsequently, 450 mL of ethyl acetate was added. The mixture was vigorously shaken for 10 min and allowed to stand until phase separation occurred, resulting in two layers: an upper organic phase (ethyl acetate) and a lower aqueous phase. The ethyl acetate phase was carefully collected in an Erlenmeyer flask. To ensure complete extraction, the aqueous layer was re-extracted three times with fresh 450 mL portions of ethyl acetate. All organic phases were combined and transferred to a round-bottom flask. The solvent was then evaporated at 50°C for 2 h using a rotary evaporator (BUCHI). The resulting concentrate was dried at 50°C for 24 h in a ventilated drying oven (MANESTY-PETERI SLW750). The final yield was 10.34%. The dried fraction was stored at −4°C until further use.

### 2.5. Cytotoxicity Evaluation of *Plectranthus glandulosus* Leaves' Aqueous Extract, Hydro-Ethanolic Extract, and Ethyl Acetate Fraction

#### 2.5.1. Cell Culture

Vero and RAW 264.7 cells were cultured according to ATCC protocols for each cell line [30, 31]. African green monkey normal kidney (Vero) cells (ATCC CRL-1586) and murine macrophage (RAW 264.7) cells (ATCC TIB-71) were grown in T-25 flasks in DMEM supplemented with 100 IU/mL penicillin, 100 μg/mL streptomycin, 10% heat-inactivated fetal bovine serum (HI-FBS), and 0.2% (w/v) NaHCO_3_. Cells were maintained at 37°C in a humidified 5% CO_2_ atmosphere. The medium was renewed every 72 h, and cell growth was monitored using an Etaluma 520 inverted microscope until a monolayer formed. Once cultures reached approximately 90% confluence, cells were trypsinized with 0.05% Trypsin-EDTA, centrifuged at 1800 rpm for 5 min, and the resulting pellet was resuspended in fresh culture medium.

#### 2.5.2. Cytotoxicity Assay

The cytotoxicity of the plant samples in cell lines was assessed following the method described by Bowling et al. [32]. Briefly, 10^4^ cells/well were seeded in 100 μL of medium in 96-well culture plates and incubated overnight at 37°C in 5% CO_2_ to allow cell adhesion. The following day, the spent medium was discarded and replaced with fresh medium. Serial dilutions of the extracts and the fraction (12.5, 25, 50, 100, and 200 μg/mL, 10 μL per well) were added, and the plates were incubated for 48 h under the same conditions. After incubation, 10 μL of resazurin solution (0.15 mg/mL in sterile PBS) was added to each well, and the plates were further incubated for 4 h. Fluorescence, which indicates cell viability, was measured using a Magellan Infinite M200 fluorescence multi-well plate reader at excitation and emission wavelengths of 530 and 590 nm, respectively. Wells containing untreated cells served as the 100% growth control. Cell viability (%) was calculated by dividing the mean fluorescence of treated wells by that of control wells at each concentration and multiplying by 100 [33]:

(1) $$\begin{array}{c} \text{Percentage of cell viability}=\left[\frac{\Delta \text{ Absorbance treated cells}}{\Delta \text{ Absorbance untreated cells}}\right]\times 100. \end{array}$$

### 2.6. Animals and Housing

Female Wistar rats (weighing 209–213 g and approximately 6 months old) were obtained from the Institute of Medical Research and Medicinal Plants Studies (IMPM), Yaoundé, Cameroon, for use in acute and subacute toxicity studies. Before group allocation, all animals were weighed and stratified according to body weight to ensure balanced distribution across experimental groups. Tail numbers were assigned based on weight, and the rats were then randomly allocated into the different groups within each weight block to minimize selection bias. In accordance with the 3 Rs principle (Replacement, Reduction, and Refinement), the number of animals was minimized to the lowest required to obtain statistically valid results, with *n* = 5 rats per group for both acute and subacute toxicity studies. According to OECD Guideline 425 [34], females tend to be slightly more sensitive than males, ensuring that potential toxic effects are not underestimated.

The animals were housed in stainless steel wire mesh cages, placed in a cage support cart with tray holders underneath for feces collection. They were kept in a well-ventilated room under a 12-h light/dark cycle, with free access to clean tap water and standard rat feed. Animals were allowed to acclimate for 1 week before the start of the experiment. All animal procedures were conducted according to established principles of animal welfare and the guidelines in the 8th edition (2011) of the Guide for the Care and Use of Laboratory Animals. The study received ethical approval from the Joint Institutional Review Board for Animal and Human Bioethics (JIRB) under Ethical Clearance Reference No: BTC-JIRB2024-098.

### 2.7. Acute Toxicity Evaluation of *Plectranthus glandulosus* Leaves' Aqueous Extract, Hydro-Ethanolic Extract, and Ethyl Acetate Fraction

The acute toxicity of the aqueous extract, hydro-ethanolic extract, and ethyl acetate fraction of *P. glandulosus* leaves was assessed according to OECD Guideline 425 [34]. A total of 20 rats were randomly divided into four groups of five animals each (Groups I–IV). They were fasted for 12 h with free access to water and weighed before oral administration. Group I, the control group, received distilled water (10 mL/kg) orally. Groups II, III, and IV were administered a single oral dose of 5000 mg/kg body weight of the aqueous extract, the hydro-ethanolic extract, and the ethyl acetate fraction, respectively. Following dosing, food was withheld for 3 h. Each animal was observed for signs of toxicity for 5 min immediately after dosing, then every 15 min for the first 4 h, every 30 min during the next 6 h, and once daily for the next 48 h. Observations continued for up to 14 days to detect any long-term, potentially fatal toxic effects. Toxicity signs, including tremors, diarrhea, salivation, convulsions, respiratory distress, changes in food and water intake, sleep patterns, skin, fur, eye color, and mucous membrane alterations, were recorded. Other observed signs included itching, excessive grooming, repetitive circling, self-mutilation, coma, and death.

#### 2.7.1. Evaluation of Food and Water Intake in the Acute Toxicity Test

Food and water intake were monitored daily. Briefly, the daily food supply was weighed, and the amount remaining the following day was recorded. The difference was calculated as the daily food intake. A similar procedure was used for water consumption. No water was spilled because standard sipper bottles with ball-bearing valves were used; these valves prevent leakage unless actively licked by the animal, ensuring accurate measurement of water intake. For food consumption, spilled food was collected, separated from feces, and dried before weighing [35].

(2) $$\begin{array}{c} \text{Total food intake }\left(\text{TF}\right)=\text{Initial food weight }\left(F_{0}\right)-\text{Leftover food weight }\left(F_{1}\right)-\text{Spilled food weight }\left(F_{2}\right),\\ \text{Total water intake }\left(\text{TW}\right)=\text{Initial water volume }\left(V_{0}\right)-\text{Leftover water volume }\left(V_{1}\right). \end{array}$$

#### 2.7.2. Evaluation of Body Weights and Organ Relative Mass in the Acute Toxicity Test

Body weights were recorded on Days 0, 7, and 14. On Day 14, following the final weighing, the animals were anesthetized via intraperitoneal injection with ketamine (80 mg/kg) and diazepam (10 mg/kg). The animals were then sacrificed by cervical dislocation. The liver, kidneys, spleen, and heart were removed, weighed, and examined macroscopically. The relative mass of these organs was calculated using the following formula [36]:

(3) $$\begin{array}{c} \text{Organ relative mass}=\left[\frac{\text{Mass of the organ}}{\text{Mass of the animal}}\right]\times 100. \end{array}$$

### 2.8. Subacute Toxicity Evaluation of *Plectranthus glandulosus* Leaves' Aqueous Extract, Hydro-Ethanolic Extract, and Ethyl Acetate Fraction

The subacute toxicity study was conducted in accordance with OECD Guideline 407 [37]. A total of 50 rats were randomly assigned to 10 groups of five animals each. Group I served as the control group and received a daily oral gavage of distilled water (10 mL/kg). Groups II–IV received the aqueous extract, Groups V–VII received the hydro-ethanolic extract, and Groups VIII–X received the ethyl acetate fraction. Rat doses were extrapolated from the human dose as described by Zouheira et al. [28] using the method of Reagan-Shaw et al. [38]. Prior pharmacological evaluations at 100, 200, and 400 mg/kg showed no adverse effects in rats. Based on these results, a low dose of 250 mg/kg was selected to slightly exceed the therapeutic range, aiming to identify a potential no-observed-adverse-effect level (NOAEL). The mid-dose of 500 mg/kg was chosen to assess dose-related effects and bridge the gap between low and high exposures. The high dose of 1000 mg/kg was selected as the limit dose recommended by OECD Guideline 407 [37] to evaluate potential toxicity at the upper exposure range. Accordingly, each extract was administered orally at doses of 250, 500, and 1000 mg/kg once daily for 28 consecutive days. During the study period, all animals were observed daily for clinical signs of toxicity, including tremors, diarrhea, salivation, convulsions, respiratory distress, changes in food and water intake, and sleep disturbances. Observations also included alterations in skin, fur, and eye coloration, as well as mucous membrane changes. Other signs included itching, excessive grooming, repetitive circling, self-mutilation, walking backward, coma, and death.

#### 2.8.1. Evaluation of Food, Water Intake, and Weight Gain in the Subacute Toxicity Test

Food and water intake were monitored daily, as described in the acute toxicity study, and summarized on a weekly basis. Body weights of the animals were recorded weekly throughout the 28-day study. At the end of the experiment, weight gain for each group was calculated using the following formula [36]:

(4) $$\begin{array}{c} \text{Animal weight gain }\left(\text{WG}\right)=\text{Final body weight on }D\text{ay }28\;\left(W_{28}\right)-\text{Initial body weight on }D\text{ay }1\;\left(W_{1}\right). \end{array}$$

#### 2.8.2. Evaluation of Digestibility Index (DI) and Food and Water Consumption Index in the Subacute Toxicity Test

The DI, representing the proportion of a given food that is absorbed and utilized by the body after digestion, was evaluated from daily food intake and the corresponding fecal mass. Excreta from each group were collected, dried at 20°C for 24 h, and then weighed. The weekly DI [39] and the food and water consumption indices [36] were calculated using the following formulas:

(5) $$\begin{array}{c} \text{DI}=\left[\frac{\text{Quantity of food intake}-\text{Mass of Feces}}{\text{Quantity of food intake}}\right]\times 100,\\ \text{Food consumption index}=\left[\frac{\text{Quantity of food intake}}{\text{Animal body weight}}\right]\times 100,\\ \text{Water consumption index}=\left[\frac{\text{Quantity of water intake}}{\text{Animal body weight}}\right]\times 100. \end{array}$$

#### 2.8.3. Blood and Organ Collection Samples in the Subacute Toxicity Test

At the end of the 28-day treatment period, the animals were fasted for 12 h while still having free access to drinking water. On Day 29, the rats were weighed, and blood samples were collected from the retro-orbital plexus using capillary tubes. The blood was transferred into heparinized tubes for hematological analysis and into plain tubes for biochemical analysis. Following blood collection, the animals were anesthetized via intraperitoneal injection with ketamine (80 mg/kg) and diazepam (10 mg/kg). The animals were then sacrificed by cervical dislocation. After euthanasia, the animals were dissected, and the liver, kidneys, heart, and spleen were harvested. The organs were rinsed with 0.9% sodium chloride (NaCl) solution, blotted dry with absorbent paper, weighed, and preserved in 10% neutral buffered formalin for histopathological examination. Relative organ weights were calculated using the following formula [36]:

(6) $$\begin{array}{c} \text{Organ relative mass}=\left[\frac{\text{Mass of the organ}}{\text{Mass of the animal}}\right]\times 100. \end{array}$$

#### 2.8.4. Evaluation of Hematological Parameters in the Subacute Toxicity Test

Heparinized blood samples were immediately analyzed using an automated hematology analyzer to determine hematological parameters, including white blood cells (WBC), red blood cells (RBC), hemoglobin (Hb), hematocrit (HCT), mean corpuscular volume (MCV), platelets (PLT), platelet distribution width (PDW), neutrophils (NEUT), lymphocytes (LYMPH), monocytes (MONO), eosinophils (EO), and basophils (BASO).

#### 2.8.5. Evaluation of Biochemical Parameters in the Subacute Toxicity Test

Serum was separated from nonheparinized blood by centrifugation at 3000 rpm for 20 min, carefully aspirated using a micropipette, and stored in Eppendorf tubes at −20°C until analysis. Biochemical parameters, including ALT, AST, Crea, urea, TP, and CRP, were measured using commercially available colorimetric kits and a spectrophotometer.

#### 2.8.6. Histopathological Examination

After 48 h of fixation in 10% neutral buffered formalin, the liver, kidneys, heart, and spleen were dehydrated through a graded ethanol series, cleared in xylene, and embedded in paraffin wax. Tissue sections (3–5 μm thick) were obtained using a microtome and stained with hematoxylin and eosin (H&E) for routine histological evaluation. The stained sections were examined using a light microscope at 100x magnification. Histopathological evaluation was performed in a blinded manner based on standard morphological criteria. A semiquantitative scoring system was applied to assess tissue alterations where applicable, using the following scale: 0 = no lesion, 1 = minimal, 2 = mild, 3 = moderate, and 4 = severe [40].

### 2.9. Statistical Analysis

All results are expressed as mean ± standard deviation (SD). Statistical analyses were performed using GraphPad Prism Version 5.00 (GraphPad Software, Inc.). One-way analysis of variance (ANOVA) followed by Tukey's multiple comparison test was performed to assess statistical significance. Differences were considered significant at *p* < 0.05. The sample size was *n* = 3 for the cytotoxicity assay and *n* = 5 for the acute and subacute toxicity tests.

## 3. Results

### 3.1. Cytotoxicity Effect of *Plectranthus glandulosus* Leaves' Aqueous Extract, Hydro-Ethanolic Extract, and Ethyl Acetate Fraction

**Figure 1:**
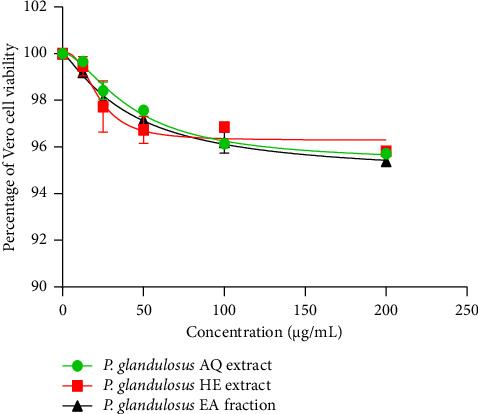
Effect of the aqueous extract, hydro-ethanolic extract, and ethyl acetate fraction of *Plectranthus glandulosus* leaves on Vero cell viability. Results are expressed as the percentage of viable cells relative to untreated controls (100% viability). Each point represents the mean of three independent experiments (*n* = 3). *P. glandulosus* AQ extract = *Plectranthus glandulosus* aqueous extract; *P. glandulosus* HE extract = *Plectranthus glandulosus* hydro-ethanolic extract; *P. glandulosus* EA fraction = *Plectranthus glandulosus* ethyl acetate fraction.

**Figure 2:**
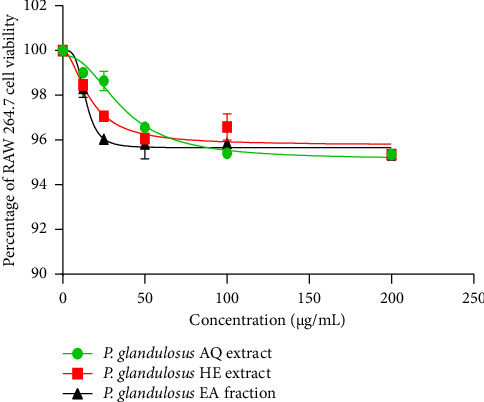
Effect of the aqueous extract, hydro-ethanolic extract, and ethyl acetate fraction of *Plectranthus glandulosus* leaves on RAW 264.7 cell viability. Results are expressed as the percentage of viable cells relative to untreated controls (100% viability). Each point represents the mean of three independent experiments (*n* = 3). *P. glandulosus* AQ extract = *Plectranthus glandulosus* aqueous extract; *P. glandulosus* HE extract = *Plectranthus glandulosus* hydro-ethanolic extract; *P. glandulosus* EA fraction = *Plectranthus glandulosus* ethyl acetate fraction.

As shown in Figure 1, all tested samples maintained Vero cell viability above 95% at the highest concentration (200 μg/mL) and above 99% at the lowest concentration (12.5 μg/mL). Similarly, in RAW 264.7 cells (Figure 2), all samples maintained cell viability above 95% at 200 μg/mL. At 12.5 μg/mL, the aqueous extract showed 99.01 ± 0.023% viability, whereas the hydro-ethanolic extract and ethyl acetate fraction showed 98.46 ± 0.23% and 98.30 ± 0.40% viability, respectively.

### 3.2. Effect of *Plectranthus glandulosus* Leaves' Aqueous Extract, Hydro-Ethanolic Extract, and Ethyl Acetate Fraction on Physical and Behavioral Signs in the Acute Toxicity Test

Acute oral administration of the aqueous extract, hydro-ethanolic extract, and ethyl acetate fraction of *Plectranthus glandulosus* leaves at 5000 mg/kg was well tolerated in rats. No clinical signs of toxicity were observed during the 14-day monitoring period. This included tremor, diarrhea, salivation, convulsions, respiratory distress, changes in feeding or drinking behavior, altered sleep patterns, and changes in skin, fur, eye color, mucous membranes, grooming behavior, motor coordination, or consciousness. These findings suggest that the tested extracts and fraction may be considered safe at 5000 mg/kg. Accordingly, the LD_50_ is estimated to be greater than 5000 mg/kg in rats.

### 3.3. Effect of *Plectranthus glandulosus* Leaves' Aqueous Extract, Hydro-Ethanolic Extract, and Ethyl Acetate Fraction on Foods and Water Consumption in the Acute Toxicity Test

**Figure 3:**
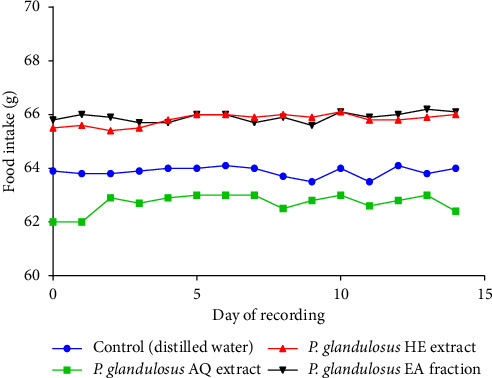
Effect of acute oral administration of the aqueous extract, hydro-ethanolic extract, and ethyl acetate fraction of *Plectranthus glandulosus* leaves on food intake in rats. Food intake is expressed in grams (g) of food consumed, *n* = 5; values are presented as the mean ± standard deviation. One-way ANOVA followed by Tukey's multiple comparison tests; no significant difference (*p* > 0.05) was observed between the control group (distilled water, 10 mL/kg) and the groups that received a single dose of 5000 mg/kg of the aqueous extract, hydro-ethanolic extract, and ethyl acetate fraction. *P. glandulosus* AQ extract = *Plectranthus glandulosus* aqueous extract; *P. glandulosus* HE extract = *Plectranthus glandulosus* hydro-ethanolic extract; *P. glandulosus* EA fraction = *Plectranthus glandulosus* ethyl acetate fraction.

**Figure 4:**
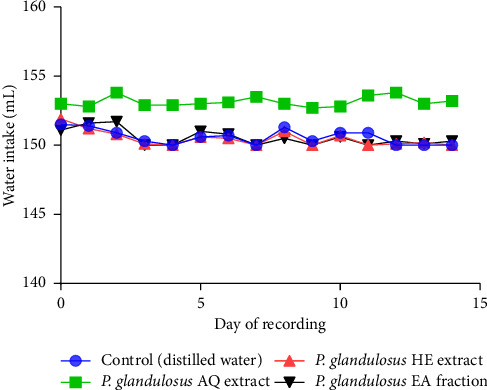
Effect of acute oral administration of the aqueous extract, hydro-ethanolic extract, and ethyl acetate fraction of *Plectranthus glandulosus* leaves on water intake in rats. Water intake is expressed in milliliters (mL) of water consumed, *n* = 5; values are presented as the mean ± standard deviation. One-way ANOVA followed by Tukey's multiple comparison tests; no significant difference (*p* > 0.05) was observed between the control group (distilled water, 10 mL/kg) and the groups that received a single dose of 5000 mg/kg of the aqueous extract, hydro-ethanolic extract, and ethyl acetate fraction. *P. glandulosus* AQ extract = *Plectranthus glandulosus* aqueous extract; *P. glandulosus* HE extract = *Plectranthus glandulosus* hydro-ethanolic extract; *P. glandulosus* EA fraction = *Plectranthus glandulosus* ethyl acetate fraction.

Administration of the aqueous extract, hydro-ethanolic extract, and ethyl acetate fraction did not significantly affect food or water consumption (*p* > 0.05). No notable changes in food or water intake were observed from Day 0 to Day 14 (Figures 3 and 4).

### 3.4. Effect of *Plectranthus glandulosus* Leaves' Aqueous Extract, Hydro-Ethanolic Extract, and Ethyl Acetate Fraction on Body Weight and Organ Relative Mass in the Acute Toxicity Test

**Figure 5:**
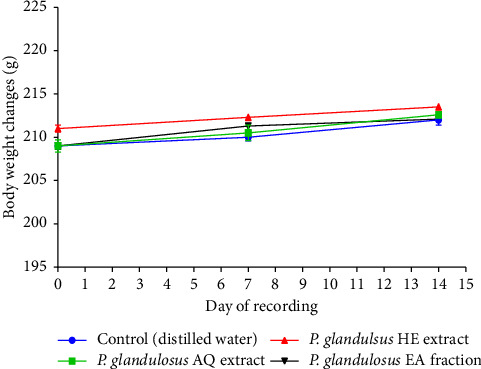
Effect of acute oral administration of the aqueous extract, hydro-ethanolic extract, and ethyl acetate fraction of *Plectranthus glandulosus* leaves on rat body weight. Rat body weight is expressed in grams (g), *n* = 5; values are presented as the mean ± standard deviation. One-way ANOVA followed by Tukey's multiple comparison tests; no significant difference (*p* > 0.05) was observed between the control group (distilled water, 10 mL/kg) and the groups that received a single dose of 5000 mg/kg of the aqueous extract, hydro-ethanolic extract, and ethyl acetate fraction. *P. glandulosus* AQ extract = *Plectranthus glandulosus* aqueous extract; *P. glandulosus* HE extract = *Plectranthus glandulosus* hydro-ethanolic extract; *P. glandulosus* EA fraction = *Plectranthus glandulosus* ethyl acetate fraction.

Animal body weight increased progressively throughout the study period. No significant difference (*p* > 0.05) in body weight gain was observed between rats treated with 5000 mg/kg of the extracts and control animals (Figure 5). Similarly, no significant differences (*p* > 0.05) in relative organ weights were observed between treated and control animals (Table 1).

### 3.5. Effect of *Plectranthus glandulosus* Leaves' Aqueous Extract, Hydro-Ethanolic Extract, and Ethyl Acetate Fraction on Organ Macroscopic Morphology in the Acute Toxicity Test

**Figure 6:**
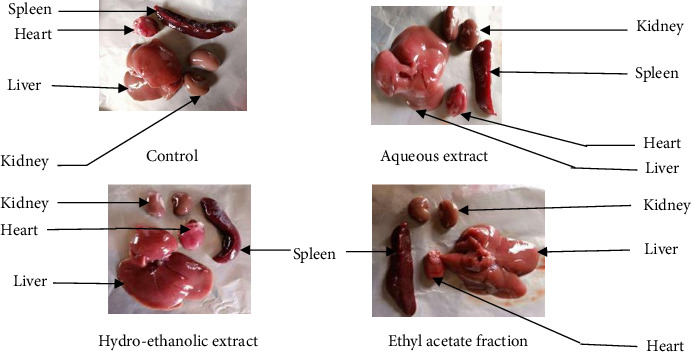
Effect of acute oral administration of the aqueous extract, hydro-ethanolic extract, and ethyl acetate fraction of *Plectranthus glandulosus* leaves on the macroscopic morphology of the spleen, kidneys, heart, and liver in rats.

Macroscopic examination of the liver, kidneys, heart, and spleen at the end of the experiment revealed no visible alterations in animals treated with the aqueous extract, hydro-ethanolic extract, or ethyl acetate fraction compared to the control group (Figure 6). All organs appeared normal in color, texture, and morphology.

### 3.6. Effect of *Plectranthus glandulosus* Leaves' Aqueous Extract, Hydro-Ethanolic Extract, and Ethyl Acetate Fraction on Physical and Behavioral Signs in the Subacute Toxicity Test

Repeated oral administration of the aqueous extract, hydro-ethanolic extract, and ethyl acetate fraction of *Plectranthus glandulosus* leaves at doses of 250, 500, and 1000 mg/kg over 28 days did not cause significant (*p* > 0.05) behavioral changes in rats. No mortality occurred during the experimental period.

### 3.7. Effect of *Plectranthus glandulosus* Leaves' Aqueous Extract, Hydro-Ethanolic Extract, and Ethyl Acetate Fraction on Food, Water Intake, and DI in the Subacute Toxicity Test

Repeated administration of the aqueous extract, hydro-ethanolic extract, and ethyl acetate fraction of *Plectranthus glandulosus* leaves at doses of 250, 500, and 1000 mg/kg over 4 weeks did not significantly affect food or water intake compared to the control group (Tables 2 and 3). Similarly, the DI did not differ significantly (*p* > 0.05) between treated and control groups throughout the experimental period (Table 4).

### 3.8. Effect of *Plectranthus glandulosus* Leaves' Aqueous Extract, Hydro-Ethanolic Extract, and Ethyl Acetate Fraction on Body Weight and Organ Relative Mass in the Subacute Toxicity Test

Table 5 shows the changes in animal body weights during the subacute oral administration study. Treatment with the aqueous extract, hydro-ethanolic extract, and ethyl acetate fraction of *Plectranthus glandulosus* at doses of 250, 500, and 1000 mg/kg did not significantly affect body weight compared to the control group over 4 weeks. Final body weight gains in treated groups were comparable to those of the control group, with no significant differences observed (*p* > 0.05).

### 3.9. Effect of *Plectranthus glandulosus* Leaves' Aqueous Extract, Hydro-Ethanolic Extract, and Ethyl Acetate Fraction on Food and Water Consumption Index in the Subacute Toxicity Test

The data presented in Tables 6 and 7 indicate that repeated oral administration of the aqueous extract, hydro-ethanolic extract, and ethyl acetate fraction of *Plectranthus glandulosus* at doses of 250, 500, and 1000 mg/kg did not significantly affect food or water consumption indices (*p* > 0.05). No statistically significant differences were observed between treated and control groups throughout the experimental period.

### 3.10. Effect of *Plectranthus glandulosus* Leaves' Aqueous Extract, Hydro-Ethanolic Extract, and Ethyl Acetate Fraction on Relative Organ Weight in the Subacute Toxicity Test

Table 8 presents the relative organ weights of animals at the end of the 28-day experimental period. The results indicate that repeated oral administration of the aqueous extract, hydro-ethanolic extract, and ethyl acetate fraction of *Plectranthus glandulosus* at doses of 250, 500, and 1000 mg/kg did not cause statistically significant differences (*p* > 0.05) in the relative weights of the liver, kidneys, heart, or spleen compared to the control group, which received distilled water (10 mL/kg).

### 3.11. Effects of *Plectranthus glandulosus* Leaves' Aqueous Extract, Hydro-Ethanolic Extract, and Ethyl Acetate Fraction on Hematological Parameters in the Subacute Toxicity Test

The hematological parameters measured in this study are presented in Table 9. Reference ranges for female Wistar rats are as follows: WBC (2.5–3.6 × 10^3^/mm^3^), RBC (5.10–8.10 × 10^6^/mm^3^), PLT (330–540 × 10^3^/mm^3^), HCT (27.30%–48.40%), HB (10.70–17.70 g/dL), MCV (50–60 fL), NEUT (20.50%–37.50%), LYMPH (59.80%–73.90%), MONO (5.60%–7.30%), EO (0.5%–4.5%), BASO (0%–0.8%), and PDW (42.8%–68.5%) [41, 42]. All hematological values for treated groups remained within these physiological reference intervals, indicating that administration of the aqueous extract, hydro-ethanolic extract, and ethyl acetate fraction of *P. glandulosus* leaves did not induce hematological toxicity.

### 3.12. Effects of *Plectranthus glandulosus* Leaves' Aqueous Extract, Hydro-Ethanolic Extract, and Ethyl Acetate Fraction on Biochemical Parameters in the Subacute Toxicity Test

The biochemical parameters evaluated in this study were not significantly altered by the oral subacute administration of the aqueous extract, hydro-ethanolic extract, and ethyl acetate fraction of *Plectranthus glandulosus* at doses of 250, 500, and 1000 mg/kg (Table 10). Reference values for ALT, AST, TP, Crea, urea, and CRP in female Wistar rats are 29.34–72.16, 62.75–126.65 IU/L, 5.20–8.20 g/dL, 0.30–0.60 mg/dL, 15–45 mg/dL, and 300–600 μg/mL, respectively [6, 43–45]. All values obtained in this study were within these reference ranges for both treated and control groups. Additionally, CRP levels, measured using a latex reagent with a sensitivity of 25 μg/mL, were 300 μg/mL in all groups, except for the 1000 mg/kg dose of the ethyl acetate fraction, which exhibited a CRP level of 350 μg/mL.

### 3.13. Effects of *Plectranthus glandulosus* Leaves' Aqueous Extract, Hydro-Ethanolic Extract, and Ethyl Acetate Fraction on Organ Histopathological Status in the Subacute Toxicity Test

**Figure 7:**
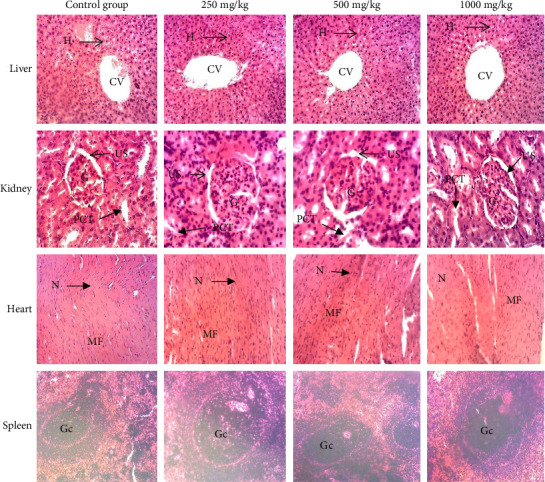
Photomicrographs (hematoxylin–eosin staining, ×100) of liver, kidney, heart, and spleen of rats after 28-day exposure to different doses of the aqueous extract. Histopathological evaluation using a semiquantitative scoring system showed a score of 0 (no lesion) for all examined tissues across treatment groups. Liver: CV = central vein, He = hepatocytes. Kidney: G = glomerulus, US = urinary space, PCT = proximal convoluted tubule. Heart: N = nucleus, MF = muscle fiber. Spleen: Gc = germinal center.

**Figure 8:**
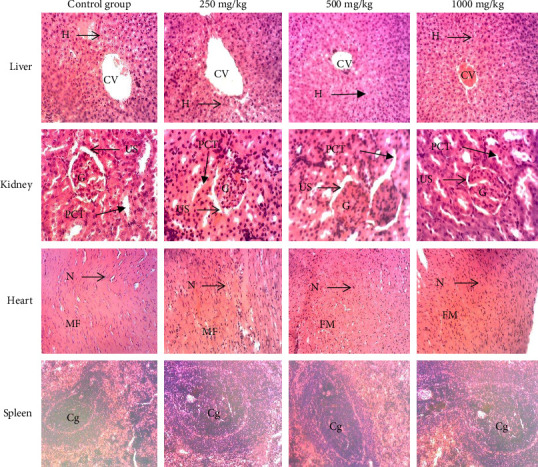
Photomicrographs (hematoxylin–eosin staining, ×100) of liver, kidney, heart, and spleen of rats after 28-day exposure to different doses of the hydro-ethanolic extract. Histopathological evaluation using a semiquantitative scoring system showed a score of 0 (no lesion) for all examined tissues across treatment groups. Liver: CV = central vein, He = hepatocytes. Kidney: G = glomerulus, US = urinary space, PCT = proximal convoluted tubule. Heart: N = nucleus, MF = muscle fiber. Spleen: Gc = germinal center.

**Figure 9:**
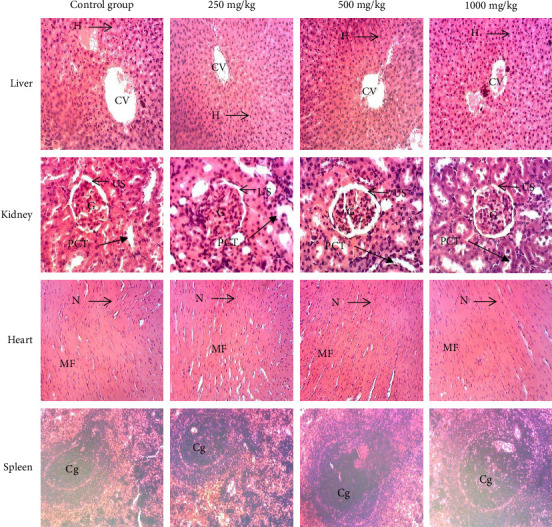
Photomicrographs (hematoxylin–eosin staining, ×100) of liver, kidney, heart, and spleen of rats after 28-day exposure to different doses of the ethyl acetate fraction. Histopathological evaluation using a semiquantitative scoring system showed a score of 0 (no lesion) for all examined tissues across treatment groups. Liver: CV = central vein, He = hepatocytes. Kidney: G = glomerulus, US = urinary space, PCT = proximal convoluted tubule. Heart: N = nucleus, MF = muscle fiber. Spleen: Gc = germinal center.

Subacute oral administration of the aqueous extract, hydro-ethanolic extract, and ethyl acetate fraction of *Plectranthus glandulosus* at doses of 250, 500, and 1000 mg/kg (Figures 7, 8, and 9) did not induce any microscopic morphological changes compared to the control group. Histopathological evaluation, using a semiquantitative scoring system (0 = no lesion, 1 = minimal, 2 = mild, 3 = moderate, and 4 = severe), assigned a score of 0 (no lesion) to all examined tissues across treatment groups. Liver sections displayed healthy hepatocytes without fatty lobulation, well-preserved cytoplasm, prominent nuclei, and intact central veins, with no signs of necrosis. Kidney tissues showed intact glomeruli, tubules, and urinary spaces. Histological analysis of the heart revealed distinct nuclei and normal myofibrillar structure with clear striations, characteristic of healthy cardiac tissue. Spleen sections exhibited well-differentiated germinal centers with both white and red pulp, without any evidence of necrosis.

## 4. Discussion

Plants have long been used to treat a wide range of illnesses. However, several previous studies have shown that some plants can exert toxic effects on various organs, potentially disrupting their physiological functions [45, 46]. In the present study, we evaluated the cytotoxicity, as well as the acute and subacute toxicity, of the aqueous extract, hydro-ethanolic extract, and ethyl acetate fraction of *Plectranthus glandulosus* leaves, a medicinal plant known for both its culinary and therapeutic applications.

### 4.1. Cytotoxicity Assessment

The cell viability assay is a crucial step in determining the nontoxic concentrations for test substances. In this study, concentrations ranging from 12.5 to 200 μg/mL of the aqueous and hydro-ethanolic extracts, as well as the ethyl acetate fraction, maintained over 95% viability in African green monkey kidney (Vero) cells and murine macrophage (RAW 264.7) cells. According to ISO 10993-5 guidelines, cell viability percentages above 80% are considered noncytotoxic [47]. These findings align with previous studies on species of the *Plectranthus* genus. Kowalczyk et al. [48] reported that extracts from *Plectranthus scutellarioides* showed only weak cytotoxicity in normal human gingival fibroblasts (HGF-1). Collectively, these results suggest that *Plectranthus* species, including *P. glandulosus*, generally exhibit favorable cytotoxicity profiles.

### 4.2. Acute and Subacute Toxicities

In the acute toxicity study of *P. glandulosus* leaves, oral administration of a single dose (5000 mg/kg) of the aqueous and hydro-ethanolic extracts, as well as the ethyl acetate fraction, did not produce any signs of toxicity or mortality in rats. Consequently, the LD_50_ was estimated to be greater than 5000 mg/kg. According to OECD criteria under the Globally Harmonized System of Classification and Labelling of Chemicals (GHS), substances with an LD_50_ between 2000 and 5000 mg/kg are classified as Category 5 or unclassified, indicating low toxicity [49]. Macroscopic examination of the liver, heart, kidneys, and spleen revealed no visible abnormalities. These findings support the safety of *P. glandulosus* extracts and the ethyl acetate fraction at the acute oral dose of 5000 mg/kg in rats. They are consistent with previous acute toxicity studies on related *Plectranthus* species, further confirming their favorable safety profile. Pillai et al. [50] reported no signs of toxicity or mortality in mice administered 2000 mg/kg of methanolic *Plectranthus amboinicus* extract. Similarly, Asiimwe et al. [51] observed no adverse effects in rats treated with 10,000 mg/kg of the aqueous extract. The aqueous extract of *Plectranthus caespitosus* also showed no toxicity at doses of 2000 and 5000 mg/kg. Collectively, these results reinforce the low acute oral toxicity of *Plectranthus* species, including *P. glandulosus*, and support their favorable therapeutic safety profile.

Repeated oral administration of the aqueous, hydro-ethanolic, and ethyl acetate extracts of *P. glandulosus* leaves at doses of 250, 500, and 1000 mg/kg for 28 consecutive days did not produce any signs of toxicity or mortality. As appetite regulates food intake and plays a key role in weight control, reductions in body weight gain or organ weights are considered sensitive indicators of toxicity following exposure to harmful substances [52]. Accordingly, changes in food and water intake, body weight gain, and relative organ weights are commonly used to assess the general health of experimental animals [53, 54]. In the present study, no significant changes in food or water consumption were observed in either the acute or subacute toxicity tests. In the subacute study, intake was normalized to body weight by calculating the total daily intake relative to body weight, termed the consumption index, allowing direct comparison between treatment groups and detection of potential toxicity-related variations. No significant differences in body weight, organ weights, or consumption indices were observed between treated and control groups, and both exhibited normal, progressive growth.

Previous analyses of *P. glandulosus* leaves demonstrated a high total phenolic content: The ethyl acetate fraction contained 139.3 ± 0.16 mg gallic acid equivalents per gram of extract (mg GAE/g), the hydro-ethanolic extract 64.28 ± 0.24 mg GAE/g, and the aqueous extract 36.80 ± 0.038 mg GAE/g [22]. Identified compounds included 7-O-methyl luteolin 5-O-β-D-glucopyranoside, chrysoeriol 5-O-β-D-glucopyranoside, 5,7-dihydroxy-3,2′,4′-trimethoxyflavone, and plectranmicin [25]. Phenol-rich extracts exhibit antioxidant and anti-inflammatory effects, support gut microbiota balance, aid in weight management, and regulate hormones, such as leptin and adiponectin, which are crucial for adipose tissue function [55, 56]. These results align with previous studies on *P. amboinicus*, where subacute oral administration of the aqueous extracts at doses of 625, 1250, and 2500 mg/kg resulted in normal body weight gains compared to controls [50]. Overall, these findings support the conclusion that members of the *Plectranthus* genus, including *P. glandulosus*, exhibit a favorable safety profile during subacute exposure.

### 4.3. Digestive Efficiency

The DI, usually expressed as a percentage, provides information on how efficiently an animal digests and absorbs its diet [57]. It is a valuable tool for assessing the impact of test extracts and fraction on digestion and absorption and may help detect potential toxicity-related effects on the gastrointestinal system. Inflammation induced by toxic substances in the gastrointestinal tract can reduce digestive efficiency and impair the absorption of both macro- and micronutrients [58]. A high DI indicates efficient digestion and absorption. In this study, no significant differences were observed between the control and treated groups. All calculated DI values were above 75%, demonstrating that the aqueous extract, hydro-ethanolic extract, and ethyl acetate fraction, at the tested doses, did not impair digestion or nutrient absorption in the animals.

### 4.4. Hematological Parameters

Hematological parameters are crucial for assessing drug-induced toxicity, providing a predictive evaluation of physiological and pathological conditions in both humans and animals [52, 53]. Reference values for female Wistar rats are as follows: WBC (2.5–3.6 × 10^3^/mm^3^), RBC (5.10–8.10 × 10^6^/mm^3^), PLT (330–540 × 10^3^/mm^3^), HCT (27.30%–48.40%), HB (10.70–17.70 g/dL), MCV (50–60 fL), NEUT (20.50%–37.50%), LYMPH (59.80%–73.90%), MONO (5.60%–7.30%), EO (0.5%–4.5%), BASO (0%–0.8%), and PDW (42.8%–68.5%) [41, 42]. Dysfunctions in these parameters may result from imbalances between blood cell production (erythropoiesis) and destruction, altered differentiation of hematopoietic stem cells, impaired Hb incorporation, or abnormal RBC morphology. Additional causes include disruptions in megakaryocyte development, bone marrow fragmentation, bowel inflammation, interference with thrombopoietin function, anemia, or polycythemia [9, 59]. In this study, hematological values in animals treated with the aqueous, hydro-ethanolic, and ethyl acetate extracts of *P. glandulosus* leaves at doses of 250, 500, and 1000 mg/kg remained within the normal physiological range and were comparable to those of the control group, suggesting that the tested extracts do not contain substances capable of disrupting hematological parameters. Phenolic compounds are known to exert hepatoprotective effects by scavenging free radicals, reducing lipid peroxidation, and enhancing the antioxidant defense system in blood cells [60]. The high phenolic content previously identified in *P. glandulosus* extracts [23, 25] likely contributed to these protective effects. Similarly, subacute administration of *P. amboinicus* at doses of 200 and 400 mg/kg did not cause hematological alterations [50], further supporting the hematological safety of *Plectranthus* species.

Serum biochemistry is essential for understanding metabolic processes and assessing the health status of various organs [61]. Evaluating hepatic and renal function is particularly important for determining the potential toxic effects of extracts and drugs on these organs [62]. Measurements of ALT, AST, TP, urea, and Crea remain reliable indicators of liver and kidney function [61].

### 4.5. Hepatic Function

Transaminases, specifically ALT and AST, are key markers of liver function. A significant elevation in their serum levels is typically associated with hepatocellular damage or disruptions in bile flow [56]. ALT, which is predominantly located in the cytoplasm of hepatocytes, serves as a more specific indicator of liver injury compared to AST [43, 61]. In this study, ALT and AST levels remained within the reference ranges for female Wistar rats (29.34–72.16 IU/L for ALT and 62.75–126.65 IU/L for AST) [42]. This consistency was observed across all treated groups receiving 250, 500, and 1000 mg/kg of *P. glandulosus* leaves' aqueous extract, hydro-ethanolic extract, and ethyl acetate fraction, as well as in the control group. These findings suggest that none of the tested extracts induced liver damage. Supporting this observation, the ethanol extract of *P. scutellarioides*, another species within the *Plectranthus* genus, has previously demonstrated hepatoprotective effects by reducing ALT and AST levels [63].

These biochemical findings were further corroborated by histological analysis, which revealed no microscopic lesions or structural alterations in the liver tissues of either treated or control animals. The absence of histopathological changes aligns with the stable transaminase levels. Together, these results reinforce the conclusion that the tested extracts and fraction do not adversely affect liver structure or function. This finding is particularly significant given the liver's critical role in detoxifying and eliminating both endogenous and exogenous substances [64]. Typically, hepatic damage is accompanied by cellular necrosis, increased lipid peroxidation, and depletion of intracellular antioxidants, such as reduced glutathione [65].

To further assess liver function, serum TP levels were evaluated, reflecting the liver's synthetic and secretory capacities. TP concentrations can be influenced by hydration status; for example, hyperproteinemia may result from dehydration due to inadequate fluid intake or excessive loss through vomiting or diarrhea [66]. In this study, TP levels in both control and treated groups remained within the normal physiological range for rats (5.20–8.20 g/dL) [42], suggesting that hepatic protein synthesis was not compromised.

The observed hepatic safety may be attributed to the phytochemical composition of the *P. glandulosus* leaf extracts and fraction. In addition to their previously reported high total phenolic content [22], the leaves contain notable levels of flavonoids (36.2%), as well as substantial amounts of terpenoids (25.6%) and saponins (18.3%) [23]. Earlier qualitative phytochemical screenings also confirmed the presence of alkaloids, tannins, and saponins in these extracts [22]. Collectively, these bioactive compounds are known to contribute to hepatoprotection, largely through their well-documented antioxidant and anti-inflammatory properties [67, 68].

### 4.6. Renal Function

Urea is a key biomarker of acute renal dysfunction and is often the earliest indicator of kidney injury to manifest [9]. During the onset of kidney disease, blood urea levels typically increase due to impaired renal clearance [61]. In adult female Wistar rats, the normal reference range for serum urea is 15–45 mg/dL [69]. In the present study, the administration of the aqueous and hydro-ethanolic extracts, as well as the ethyl acetate fraction of *P. glandulosus* leaves, at doses of 250, 500, and 1000 mg/kg, did not alter serum urea levels. All recorded values remained within the established physiological range. These results suggest that subacute oral administration of the plant extracts and fraction did not induce renal dysfunction.

In addition to urea, serum Crea is another essential indicator used to evaluate kidney function. Crea is produced endogenously and released into body fluids at a relatively constant rate. Its plasma concentration is primarily regulated through glomerular filtration, making it a standard marker for assessing the glomerular filtration rate (GFR) [43, 70]. Unlike urea, Crea levels typically rise only after substantial renal impairment has occurred, thus serving as a reliable marker of chronic kidney dysfunction [71]. The reference range for serum Crea in adult female Wistar rats is approximately 0.30–0.60 mg/dL [42]. In this study, Crea levels in both treated and control groups remained within this normal range at all administered doses of the *P. glandulosus* extracts and fraction. These consistent values indicate that the subacute treatment did not lead to chronic kidney damage.

Further supporting these biochemical findings, renal histological analyses revealed no structural abnormalities. The kidneys of treated animals, like those in the control group, exhibited normal architecture without signs of tubular damage or glomerular degeneration. This histological evidence reinforces the biochemical results, confirming the absence of renal toxicity. Altogether, the consistency of the biochemical markers and the unaltered renal histology strongly suggest that subacute exposure to the *P. glandulosus* extracts and ethyl acetate fraction does not impair kidney function.

The protective effects observed may be attributed, at least in part, to the phytochemical constituents of the plant. Phenolic compounds, previously reported in *P. glandulosus* leaves [22, 23, 25], are known to offer multiple nephroprotective benefits. These include improving renal microcirculation, protecting the tubular epithelium, reducing oxidative stress, supporting mitochondrial function, and modulating inflammatory responses in renal disorders [72].

### 4.7. Inflammation Marker

CRP, a marker of chronic inflammation, plays a major role in the development of chronic diseases [73]. Elevated CRP levels have been associated with increased risks of metabolic syndrome, cardiovascular disease, coronary heart disease, myocardial infarction, diabetes, and colon cancer [74]. CRP is also a sensitive indicator of both low-grade and acute inflammatory conditions [75] and is commonly used to assess inflammation levels and monitor the anti-inflammatory effects of drugs [76]. The CRP assay relies on an immunochemical reaction between anti-CRP antibodies bound to latex particles and CRP present in rat serum. In healthy laboratory and specific pathogen-free rats, serum CRP concentrations typically range from 300 to 600 μg/mL [38], with elevated levels causing visible agglutination of the latex particles. In this study, CRP levels were 300 μg/mL in both treated and control groups, except at the 1000 mg/kg dose of the ethyl acetate fraction, where a level of 350 μg/mL was recorded. These values fall within the normal range, indicating that repeated administration of the aqueous extract, hydro-ethanolic extract, and the ethyl acetate fraction at doses of 250, 500, and 1000 mg/kg did not induce inflammation in rats. The observed anti-inflammatory effects may be attributed to the leaf extracts and fraction, which have previously demonstrated protein denaturation inhibition, membrane-stabilizing effects, and antiproteinase activity [23].

### 4.8. Cardiovascular Safety

The heart functions as a pump that delivers blood, and consequently oxygen and other nutrients, to all tissues in the body [77]. Damage to cardiomyocytes weakens the myocardium and can impair circulation [77], while persistent rhythm disturbances may progress to heart failure or sudden death [77]. In this study, histopathological examination of heart sections from both treated and control rats revealed normal myofibrillar architecture: Striations were intact, fibers were appropriately branched, and no necrosis or other lesions were observed. These results indicate that the aqueous and hydro-ethanolic extracts, as well as the ethyl acetate fraction, of *P. glandulosus* leaves do not compromise cardiac structure or function at the tested doses. The phenolic compounds, alkaloids, tannins, terpenoids, and saponins previously identified in *P. glandulosus* leaves [22, 23, 25] are known to exert cardioprotective effects via antioxidant, anti-inflammatory, and lipid-lowering mechanisms [78].

### 4.9. Splenic Integrity

The spleen is a vital component of the immune system. It plays a key role in fighting infections by regulating the levels of WBC, RBC, and PLT [79]. Additionally, it filters the blood and removes old or damaged RBC. When the spleen is impaired, it may begin removing healthy blood cells, which can result in anemia, increased susceptibility to infections, reduced WBC counts, bleeding or bruising, and a drop in PLT numbers [79]. In this study, histological examination of spleen tissues revealed well-differentiated germinal centers, along with normal white and red pulp structures, in both the control and treated groups. This observation is consistent with the normal values obtained for RBC, WBC, and PLT counts. These findings suggest that repeated administration of the aqueous and hydro-ethanolic extracts, as well as the ethyl acetate fraction of *P. glandulosus* leaves, at doses of 250, 500, and 1000 mg/kg, does not impair spleen function. Notably, the phenolic compounds previously reported in *P. glandulosus* [22, 23, 25] are known to reduce oxidative stress and inflammation, which may prevent apoptosis in spleen cells [80], potentially contributing to the observed protective effect.

### 4.10. Potential for Human Applications

The favorable safety profile of *Plectranthus glandulosus* leaf extracts and fraction observed in this study highlights their potential for human health applications. The absence of cytotoxic, hepatotoxic, nephrotoxic, or inflammatory effects, together with preserved digestive efficiency, spleen integrity, hematological stability, and cardioprotection, supports their overall safety. Along with their previously reported antioxidant, anti-inflammatory, anti-atherogenic, antithrombotic, and anti-adipogenic properties, these findings provide a strong rationale for incorporating them into food, nutraceutical, or medicinal formulations as functional ingredients aimed at preventing or mitigating chronic diseases, such as metabolic syndrome and cardiovascular disorders. Moreover, the traditional culinary use of *P. glandulosus* as a seasoning underscores its cultural acceptability. However, future studies, including chronic toxicity and genotoxicity evaluations, as well as clinical trials, remain essential to confirm efficacy, safety, and optimal dosage before large-scale food or pharmaceutical applications.

## 5. Conclusion and Future Directions

In brief, the aqueous and hydro-ethanolic extracts, as well as the ethyl acetate fraction, showed no signs of toxicity toward Vero cells (normal kidney cells from African green monkeys) and RAW 264.7 cells (murine macrophages). All calculated cell viability percentages at the tested concentrations were above 95%. The acute toxicity study of the aqueous and hydro-ethanolic extracts, as well as the ethyl acetate fraction, at a single oral dose of 5000 mg/kg, did not produce any adverse effects on behavior or gross pathology in rats. Therefore, the LD_50_ was estimated to be greater than 5000 mg/kg. Repeated oral administration of different doses (250, 500, and 1000 mg/kg) of the aqueous and hydro-ethanolic extracts, as well as the ethyl acetate fraction, over 28 days did not cause any treatment-related side effects or mortality in rats. Analysis of acute and subacute toxicity revealed no significant changes in food and water consumption, or in body and organ weights. Furthermore, all hematological and serum biochemical parameters in the subacute toxicity test remained within the reference ranges. No macroscopic or microscopic alterations were observed in any of the examined organs in either the acute or subacute toxicity studies. These findings demonstrate the safety of *Plectranthus glandulosus* leaves and support their potential use in traditional medicine. Overall, further research on this plant represents a promising avenue for the development of novel therapeutic agents. Future studies should include chronic toxicity, genotoxicity, and clinical evaluations to fully establish its long-term safety and therapeutic potential.

### 5.1. Limitations of the Study

The sample size used in this study (*n* = 5 per group) is relatively small for subacute toxicity studies. However, this number was selected in accordance with the principles of the 3 Rs, which guide ethical animal research. Specifically, the Reduction principle encourages researchers to use the minimum number of animals. While appropriate for an initial safety assessment, larger sample sizes determined by power analysis will be employed in subsequent studies to enhance the statistical robustness and generalizability of the findings.

## Figures and Tables

**Table 1:** Effect of acute oral administration of aqueous extract, hydro-ethanolic extract, and ethyl acetate fraction of *Plectranthus glandulosus* leaves on the relative organ weight in rats.

Relative organ weight in grams				
Group	Heart	Liver	Kidneys	Spleen
Control (distilled water)	0.66 ± 0.02	4.56 ± 0.57	1.25 ± 0.02	0.54 ± 0.02
Aqueous extract	0.65 ± 0.89	4.53 ± 0.67	1.26 ± 0.12	0.53 ± 0.02
Hydro-ethanolic extract	0.67 ± 0.05	4.57 ± 0.33	1.25 ± 0.02	0.55 ± 0.02
Ethyl acetate fraction	0.67 ± 0.23	4.53 ± 0.10	1.24 ± 0.05	0.54 ± 0.02

*Note:* Rat relative organ weight is expressed in grams (g), *n* = 5; values are presented as the mean ± standard deviation. One-way ANOVA followed by Tukey's multiple comparison tests; no significant difference (*p* > 0.05) was observed between the control group (distilled water, 10 mL/kg) and the groups that received a single dose of 5000 mg/kg of the aqueous extract, hydro-ethanolic extract, and ethyl acetate fraction.

**Table 2:** Effect of subacute oral administration of the aqueous extract, hydro-ethanolic extract, and ethyl acetate fraction of *Plectranthus glandulosus* leaves on food intake in rats.

Food intake in grams				
Groups	Week 1	Week 2	Week 3	Week 4
Control (H_2_O_2_ 10 mL/kg)	58.19 ± 5.38	59.52 ± 4.37	62.71 ± 4.5	60.57 ± 4.5
AQ extract (250 mg/kg)	45.2 ± 3.35	44.10 ± 1.10	45.71 ± 1.11	45.29 ± 1.25
AQ extract (500 mg/kg)	54.71 ± 3.64	55.57 ± 2.57	54.85 ± 3.02	55.57 ± 1.99
AQ extract (1000 mg/kg)	55.14 ± 3.58	55.29 ± 3.40	56.57 ± 3.57	56.00 ± 0.58
HE extract (250 mg/kg)	54.00 ± 1.48	56.11 ± 1.22	56.57 ± 1.13	56.30 ± 1.11
HE extract (500 mg/kg)	55.21 ± 2.15	56.38 ± 3.18	57.00 ± 41.00	56.35 ± 3.41
HE extract (1000 mg/kg)	65.33 ± 1.39	66.22 ± 1.12	65.30 ± 3.11	66.15 ± 2.23
EA fraction (250 mg/kg)	64.29 ± 3.45	65.57 ± 1.38	65.71 ± 3.27	65.81 ± 2.12
EA fraction (500 mg/kg)	55.14 ± 3.85	56.17 ± 1.14	54.95 ± 3.34	55.11 ± 2.14
EA fraction (1000 mg/kg)	52.11 ± 1.10	52.27 ± 1.53	53.22 ± 3.23	53.28 ± 1.17

*Note:* Food intake is expressed in grams (g) of food consumed, *n* = 5; values are presented as the mean ± standard deviation. One-way ANOVA followed by Tukey's multiple comparison tests; no significant difference (*p* > 0.05) was observed between the control group (distilled water, 10 mL/kg) and the groups that received repeated administration of different doses (250, 500, and 1000 mg/kg) of the aqueous extract, hydro-ethanolic extract, and ethyl acetate fraction. Control group = distilled water (10 mL/kg); AQ extract = aqueous extract; HE extract = hydro-ethanolic extract; EA fraction = ethyl acetate fraction.

**Table 3:** Effect of subacute oral administration of the aqueous extract, hydro-ethanolic extract, and ethyl acetate fraction of *Plectranthus glandulosus* leaves on water intake in rats.

Water intake in mL				
Groups	Week 1	Week 2	Week 3	Week 4
Control	150.50 ± 0.38	150.60 ± 0.37	150.32 ± 4.5	150.57 ± 4.5
AQ extract (250 mg/kg)	145.2 ± 3.25	144.10 ± 2.10	145.71 ± 0.11	145.29 ± 3.22
AQ extract (500 mg/kg)	154.50 ± 3.64	155.5 ± 2.57	154.8 ± 3.02	155.5 ± 1.29
AQ extract (1000 mg/kg)	150.03 ± 3.58	150.11 ± 1.30	150.13 ± 1.12	151.00 ± 0.58
HE extract (250 mg/kg)	156.00 ± 2.31	156.01 ± 0.13	155.17 ± 1.21	157.10 ± 2.00
HE extract (500 mg/kg)	154.11 ± 1.15	155.13 ± 1.12	154.00 ± 1.10	155.15 ± 1.23
HE extract (1000 mg/kg)	155.11 ± 1.12	156.02 ± 0.13	155.11 ± 2.01	156.04 ± 1.11
EA fraction (250 mg/kg)	144.11 ± 1.12	145.03 ± 1.21	145.01 ± 1.08	145.21 ± 0.15
EA fraction (500 mg/kg)	151.21 ± 1.35	153.12 ± 1.13	152.13 ± 1.13	153.01 ± 1.11
EA fraction (1000 mg/kg)	152.02 ± 0.10	150.17 ± 1.23	152.12 ± 1.03	153.03 ± 1.13

*Note:* Water intake is expressed in milliliters (mL) of water consumed, *n* = 5; values are presented as the mean ± standard deviation. One-way ANOVA followed by Tukey's multiple comparison tests; no significant difference (*p* > 0.05) was observed between the control group (distilled water, 10 mL/kg) and the groups that received repeated administration of different doses (250, 500, and 1000 mg/kg) of the aqueous extract, hydro-ethanolic extract, and ethyl acetate fraction. Control group = distilled water (10 mL/kg); AQ extract = aqueous extract; HE extract = hydro-ethanolic extract; EA fraction = ethyl acetate fraction.

**Table 4:** Effect of subacute oral administration of the aqueous extract, hydro-ethanolic extract, and ethyl acetate fraction of *Plectranthus glandulosus* leaves on the digestibility index in rats.

Digestibility index (%)				
Groups	Week 1	Week 2	Week 3	Week 4
Control (H_2_0, 10 mL/kg)	82.81	81.51	84.05	83.49
AQ extract (250 mg/kg)	77.87	79.59	80.31	79.02
AQ extract (500 mg/kg)	82.63	82.00	83.59	83.80
AQ extract (1000 mg/kg)	83.22	82.36	84.09	83.92
HE extract (250 mg/kg)	83.33	83.96	80.55	82.23
HE extract (500 mg/kg)	81.88	82.26	84.21	82.25
HE extract (1000 mg/kg)	84.69	84.52	84.68	85.26
EA fraction (250 mg/kg)	84.83	84.74	83.25	83.28
EA fraction (500 mg/kg)	83.67	80.41	83.16	83.66
EA fraction (1000 mg/kg)	80.80	82.78	81.67	83.10

*Note:* Digestibility index is expressed as the percentage of food digested (%), *n* = 5. One-way ANOVA followed by Tukey's multiple comparison tests; no significant difference (*p* > 0.05) was observed between the control group (distilled water, 10 mL/kg) and the groups that received repeated administration of different doses (250, 500, and 1000 mg/kg) of the aqueous extract, hydro-ethanolic extract, and ethyl acetate fraction. Control group = distilled water (10 mL/kg); AQ extract = aqueous extract; HE extract = hydro-ethanolic extract; EA fraction = ethyl acetate fraction.

**Table 5:** Effect of subacute oral administration of the aqueous extract, hydro-ethanolic extract, and ethyl acetate fraction of *Plectranthus glandulosus* leaves on rat body weight.

Rat body weight in grams						
Groups	Day 1	Day 7	Day 14	Day 21	Day 28	Total weight gain
Control	213.00 ± 0.41	218.40 ± 3.11	223.00 ± 4.13	228.20 ± 5.6	233.60 ± 5.01	20.60 ± 3.06
AQ extract 250 mg/kg	209.00 ± 0.19	214.53 ± 3.00	219.00 ± 3.30	223.98 ± 2.17	229.88 ± 1.17	20.88 ± 3.10
AQ extract 500 mg/kg	211.60 ± 3.57	216.14 ± 5.23	221.20 ± 1.32	226.60 ± 5.90	231.80 ± 3.02	20.20 ± 4.04
AQ extract 1000 mg/kg	210.20 ± 3.80	215.40 ± 3.72	220.98 ± 2.70	224.80 ± 4.40	230.25 ± 1.15	20.05 ± 2.52
HE extract 250 mg/kg	210.60 ± 5.92	215.18 ± 5.50	220.96 ± 2.50	225.51 ± 3.90	231.20 ± 5.15	20.60 ± 3.15
HE extract 500 mg/kg	209.60 ± 3.78	215.29 ± 3.00	220.60 ± 6.18	225.40 ± 1.92	230.55 ± 2.40	20.95 ± 1.36
HE extract 1000 mg/kg	212.80 ± 0.83	217.58 ± 4.36	222.92 ± 1.36	226.23 ± 3.32	232.80 ± 6.18	20.00 ± 2.39
EA fraction 250 mg/kg	209.60 ± 3.67	214.97 ± 5.34	219.20 ± 14.00	224.23 ± 0.65	229.98 ± 5.16	20.38 ± 3.91
EA fraction 500 mg/kg	209.40 ± 4.82	214.29 ± 5.02	219.60 ± 6.18	224.40 ± 3.20	229.60 ± 2.40	20.20 ± 2.98
EAF fraction 1000 mg/kg	211.60 ± 3.50	216.82 ± 5.40	221.20 ± 5.72	226.20 ± 5.50	231.60 ± 5.60	20.00 ± 1.81

*Note:* Rat body weight is expressed in grams (g), *n* = 5; values are presented as the mean ± standard deviation. One-way ANOVA followed by Tukey's multiple comparison tests; no significant difference (*p* > 0.05) was observed between the control group (distilled water, 10 mL/kg) and the groups that received repeated administration of different doses (250, 500, and 1000 mg/kg) of the aqueous extract, hydro-ethanolic extract, and ethyl acetate fraction. Control group = distilled water (10 mL/kg); AQ extract = aqueous extract; HE extract = hydro-ethanolic extract; EA fraction = ethyl acetate fraction.

**Table 6:** Effect of subacute oral administration of the aqueous extract, hydro-ethanolic extract, and ethyl acetate fraction of *Plectranthus glandulosus* leaves on food consumption index in rats.

Food consumption index (%)				
Groups	Week 1	Week 2	Week 3	Week 4
Control (H_2_O, 10 mL/kg)	0.26	0.26	0.27	0.25
AQ extract (250 mg/kg)	0.21	0.20	0.20	0.19
AQ extract (500 mg/kg)	0.25	0.25	0.24	0.24
AQ extract (1000 mg/kg)	0.25	0.25	0.25	0.24
HE extract (250 mg/kg)	0.25	0.25	0.25	0.24
HE extract (500 mg/kg)	0.25	0.25	0.25	0.24
HE extract (1000 mg/kg)	0.30	0.29	0.28	0.28
EA fraction (250 mg/kg)	0.29	0.29	0.29	0.28
EA fraction (500 mg/kg)	0.25	0.25	0.24	0.24
EA fraction (1000 mg/kg)	0.24	0.23	0.23	0.23

*Note:* Food consumption index is expressed as the percentage (%) of food intake relative to the animal's body weight, *n* = 5. One-way ANOVA followed by Tukey's multiple comparison tests; no significant difference (*p* > 0.05) was observed between the control group (distilled water, 10 mL/kg) and the groups that received repeated administration of different doses (250, 500, and 1000 mg/kg) of the aqueous extract, hydro-ethanolic extract, and ethyl acetate fraction. Control group = distilled water (10 mL/kg); AQ extract = aqueous extract; HE extract = hydro-ethanolic extract; EA fraction = ethyl acetate fraction.

**Table 7:** Effect of subacute oral administration of the aqueous extract, hydro-ethanolic extract, and ethyl acetate fraction of *Plectranthus glandulosus* leaves on water consumption index in rats.

Water consumption index (%)				
Groups	Week 1	Week 2	Week 3	Week 4
Control	0.68	0.67	0.65	0.64
AQ extract (250 mg/kg)	0.67	0.65	0.65	0.63
AQ extract (500 mg/kg)	0.71	0.70	0.68	0.67
AQ extract (1000 mg/kg)	0.69	0.67	0.66	0.65
HE extract (250 mg/kg)	0.72	0.70	0.68	0.67
HE extract (500 mg/kg)	0.71	0.70	0.68	0.67
HE extract (1000 mg/kg)	0.72	0.70	0.68	0.67
EA fraction (250 mg/kg)	0.66	0.65	0.64	0.62
EA fraction (500 mg/kg)	0.70	0.69	0.67	0.66
EA fraction (1000 mg/kg)	0.70	0.67	0.67	0.66

*Note:* Water consumption index is expressed as the percentage (%) of water intake relative to the animal's body weight, *n* = 5. One-way ANOVA followed by Tukey's multiple comparison tests; no significant difference (*p* > 0.05) was observed between the control group (distilled water, 10 mL/kg) and the groups that received repeated administration of different doses (250, 500, and 1000 mg/kg) of the aqueous extract, hydro-ethanolic extract, and ethyl acetate fraction. Control group = distilled water (10 mL/kg); AQ extract = aqueous extract; HE extract = hydro-ethanolic extract; EA fraction = ethyl acetate fraction.

**Table 8:** Effect of subacute oral administration of the aqueous extract, hydro-ethanolic extract, and ethyl acetate fraction of *Plectranthus glandulosus* leaves on rat relative organ weight.

**Relative organ weight (%)**				
	**Heart**	**Liver**	**Kidneys**	**Spleen**

Control	0.73 ± 0.05	5.82 ± 1.31	1.62 ± 0.11	0.53 ± 0.24
AQ extract 250 mg/kg	0.72 ± 0.06	5.53 ± 0.54	1.50 ± 0.13	0.56 ± 0.23
AQ extract 500 mg/kg	0.73 ± 0.05	5.56 ± 0.51	1.65 ± 0.19	0.57 ± 0.16
AQ extract 1000 mg/kg	0.71 ± 0.04	5.42 ± 0.41	1.23 ± 0.02	0.51 ± 0.10
HE extract 250 mg/kg	0.72 ± 0.07	5.43 ± 0.03	1.40 ± 0.04	0.50 ± 0.03
HE extract 500 mg/kg	0.73 ± 0.01	5.46 ± 0.11	1.55 ± 0.03	0.54 ± 0.04
HE extract 1000 mg/kg	0.71 ± 0.06	5.62 ± 0.42	1.23 ± 0.03	0.52 ± 0.01
EA fraction 250 mg/kg	0.72 ± 0.09	5.46 ± 0.97	1.41 ± 0.25	0.52 ± 0.09
EA fraction 500 mg/kg	0.73 ± 0.05	5.20 ± 0.65	1.21 ± 0.06	0.55 ± 0.03
EA fraction 1000 mg/kg	0.72 ± 0.04	5.06 ± 0.47	1.67 ± 0.19	0.56 ± 0.23

*Note:* Rat relative organ weight is expressed as a percentage (%) of the animal's body weight, *n* = 5; values are presented as the mean ± standard deviation. One-way ANOVA followed by Tukey's multiple comparison tests; no significant difference (*p* > 0.05) was observed between the control group (distilled water, 10 mL/kg) and the groups that received repeated administration of different doses (250, 500, and 1000 mg/kg) of the aqueous extract, hydro-ethanolic extract, and ethyl acetate fraction. Control group = distilled water (10 mL/kg); AQ extract = aqueous extract; HE extract = hydro-ethanolic extract; EA fraction = ethyl acetate fraction.

**Table 9:** Effect of subacute oral administration of the aqueous extract, hydro-ethanolic extract, and ethyl acetate fraction of *Plectranthus glandulosus* leaves on hematological parameters in rats.

Parameter	Reference values [41, 42]	Control	AQ 250 mg/kg	AQ 500 mg/kg	AQ 1000 mg/kg	HE 250 mg/kg	HE 500 mg/kg	HE 1000 mg/kg	EA 250 mg/kg	EA 500 mg/kg	EA 1000 mg/kg
WBC (×10^3^/mm^3^)	**2.5–3.6**	7.94 ± 1.29	8.00 ± 1.10	8.00 ± 1.32	7.90 ± 0.70	8.00 ± 0.60	7.91 ± 0.40	7.23 ± 0.20	8.00 ± 0.80	7.11 ± 0.20	7.95 ± 0.93
RBC (×10^6^/mm^3^)	**5.10–8.10**	3.57 ± 0.79	3.50 ± 0.50	2.55 ± 0.42	3.00 ± 0.50	2.40 ± 0.60	3.00 ± 0.30	3.30 ± 0.40	3.20 ± 0.80	3.40 ± 0.10	3.38 ± 0.35
HB (g/dL)	**10.70–17.70**	12.20 ± 1.10	12.20 ± 0.20	12.20 ± 0.20	12.10 ± 0.30	12.70 ± 0.50	12.40 ± 0.60	12.40 ± 0.40	12.40 ± 1.40	12.30 ± 0.50	12.40 ± 0.62
HCT (%)	**27.30–48.40**	41.70 ± 3.70	33.20 ± 3.80	28.20 ± 3.80	33.10 ± 0.40	44.10 ± 2.40	42.70 ± 2.90	29.60 ± 0.40	38.40 ± 4.50	27.80 ± 1.20	44.53 ± 1.25
MCV (fL)	**50–60**	55.20 ± 1.20	61.50 ± 0.20	58.50 ± 0.20	52.70 ± 1.20	54.60 ± 0.60	58.20 ± 7.80	53.70 ± 2.80	56.40 ± 1.50	59.30 ± 0.80	57.77 ± 3.40
PLT (×10^3^/mm^3^)	**330–540**	490.70 ± 0.40	552.90 ± 1.50	393.70 ± 1.30	491.30 ± 11.00	441.30 ± 0.15	421.00 ± 0.10	522.00 ± 1.70	392.30 ± 1.50	420.67 ± 0.19	528.13 ± 1.63
PDW (%)	**42.8–68.5**	17.90 ± 0.50	18.00 ± 0.30	18.00 ± 0.30	18.40 ± 0.20	18.50 ± 0.40	18.40 ± 1.50	18.40 ± 0.40	17.40 ± 0.30	18.17 ± 0.12	17.80 ± 0.20
NEUT (%)	**20.50–37.50**	24.80 ± 1.60	32.80 ± 0.80	27.80 ± 0.80	25.70 ± 1.10	34.00 ± 0.50	31.50 ± 0.50	33.60 ± 2.80	29.80 ± 2.30	27.43 ± 1.53	29.17 ± 0.50
LYMPH (%)	**59.80–73.90**	60.60 ± 0.80	62.90 ± 1.80	65.90 ± 1.80	71.70 ± 0.30	66.90 ± 9.70	64.60 ± 1.00	65.10 ± 0.70	70.90 ± 0.80	72.57 ± 1.85	71.40 ± 1.13
MONO (%)	**5.60–7.30**	7.10 ± 0.20	7.60 ± 0.70	7.60 ± 0.70	7.10 ± 0.40	7.20 ± 0.90	7.40 ± 0.30	7.50 ± 0.20	7.30 ± 0.30	7.50 ± 0.20	7.57 ± 0.42
EO (%)	**0.5–4.5**	1.50 ± 0.40	1.60 ± 0.20	1.30 ± 0.20	1.42 ± 0.10	1.56 ± 0.20	1.39 ± 0.20	1.57 ± 0.30	1.34 ± 0.40	1.63 ± 0.12	1.67 ± 0.49
BASO (%)	**0–0.8**	0.4 ± 0.20	0.43 ± 0.50	0.39 ± 0.12	0.38 ± 0.13	0.40 ± 0.22	0.44 ± 0.50	0.46 ± 0.90	0.37 ± 0.33	0.48 ± 0.23	0.44 ± 0.54

*Note:* Each value represents the mean ± standard deviation, *n* = 5 animals. Control group = distilled water (10 mL/kg); AQ extract = aqueous extract; HE extract = hydro-ethanolic extract; EA fraction = ethyl acetate fraction; HB: hemoglobin; HCT: hematocrit, PLT: platelets; NEUT: neutrophils; LYMPH: lymphocytes; MONO: monocytes; EO: eosinophils; BASO: basophils. Bold values represent the reference (normal) values of WBC, RBC, HB, HCT, MCV, PLT, PDW, NEUT, LYMPH, MONO, EO, and BASO.

Abbreviations: MCV, mean corpuscular volume; PDW, platelet distribution width; RBC, red blood cells; WBC, white blood cells.

**Table 10:** Effect of subacute oral administration of the aqueous extract, hydro-ethanolic extract, and ethyl acetate fraction of *Plectranthus glandulosus* leaves on biochemical parameters in rats.

	ALAT (U/L)	AST (U/L)	TP (g/L)	Crea (mg/dL)	Urea (mg/dL)	CRP (μg/mL)
Reference values [6, 43–45]	**29.34–72.16**	**62.75–126.65**	**52.0–82.0**	**0.30–0.60**	**15–45**	**300–600**
Control	64.60 ± 10.25	120.69 ± 9.56	65.2 ± 3.20	0.56 ± 0.33	21.62 ± 0.73	300
AQ 250 mg/kg	56.11 ± 5.79	122.55 ± 9.83	66.57 ± 4.23	0.57 ± 0.20	20.15 ± 1.41	300
AQ 500 mg/kg	55.55 ± 5.86	118.98 ± 6.60	64.44 ± 1.88	0.58 ± 0.12	20.22 ± 2.64	300
AQ 1000 mg/kg	62.76 ± 5.71	112.15 ± 7.67	64.45 ± 2.28	0.56 ± 0.06	21.22 ± 2.26	300
HE 250 mg/kg	59.71 ± 7.34	121.10 ± 7.34	64.91 ± 4.44	0.57 ± 0.11	21.50 ± 2.14	300
HE 500 mg/kg	58.85 ± 4.93	124.64 ± 1.87	65.12 ± 1.97	0.56 ± 0.30	19.98 ± 3.44	300
HE 1000 mg/kg	69.20 ± 6.46	123.85 ± 2.90	65.80 ± 5.70	0.56 ± 0.05	20.35 ± 4.16	300
EA 250 mg/kg	71.15 ± 8.69	120.10 ± 2.24	65.20 ± 6.75	0.57 ± 0.33	21.27 ± 3.27	300
EA 500 mg/kg	65.11 ± 8.98	123.95 ± 1.37	66.83 ± 3.57	0.58 ± 0.12	20.88 ± 7.50	300
EA 1000 mg/kg	63.90 ± 10.22	115.15 ± 1.94	65.64 ± 3.20	0.58 ± 0.32	20.74 ± 3.27	350

*Note:* Each value represents the mean ± standard deviation, *n* = 5 animals. Control group = distilled water (10 mL/kg); AQ extract = aqueous extract; HE extract = hydro-ethanolic extract; EA fraction = ethyl acetate fraction; ALAT = alanine aminotransferase; AST = aspartate aminotransferase; Crea = creatinine. Bold values represent the reference (normal) values of ALAT (U/L), AST (U/L), total protein (g/L), creatinine (mg/dL), urea (mg/dL), and CRP (μg/mL).

Abbreviations: CRP, C-reactive protein; TP, total protein.

## Data Availability

All the data used to support the findings of this study are included within the article.
